# Microvessel rupture induced by high-intensity therapeutic ultrasound—a study of parameter sensitivity in a simple in vivo model

**DOI:** 10.1186/s40349-017-0082-2

**Published:** 2017-03-02

**Authors:** Yeonho Kim, Marjan Nabili, Priyanka Acharya, Asis Lopez, Matthew R. Myers

**Affiliations:** 1 Preclinical Studies Core, Center for Neuroscience and Regenerative Medicine, Uniformed Services University of the Health Sciences, 4301 Jones Bridge Road, Bethesda, MD 20814 USA; 2 Division of Radiological Health, Office of In-Vitro Diagnostics and Radiological Health, Center for Devices and Radiological Health, U. S. Food and Drug Administration, 10903 New Hampshire Avenue, Building 66, Room 4311, Silver Spring, MD 20993 USA; 3 Department of Chemical and Biomolecular Engineering, University of Maryland College Park, 4418 Stadium Drive, College Park, MD 20742 USA; 4 Bioinnovation PhD Program, School of Science and Engineering, Tulane University, 6823 St. Charles Avenue, Lindy Boggs Center, Room 440, New Orleans, LA 70118 USA; 5 Division of Applied Mechanics, Office of Science and Engineering Laboratories, Center for Devices and Radiological Health, U. S. Food and Drug Administration, 10903 New Hampshire Avenue, Building 62, Room 2231, Silver Spring, MD 20993 USA

**Keywords:** Vessel rupture, Therapeutic ultrasound, Transcranial ultrasound, High-intensity focused ultrasound, Transcranial ultrasound

## Abstract

**Background:**

Safety analyses of transcranial therapeutic ultrasound procedures require knowledge of the dependence of the rupture probability and rupture time upon sonication parameters. As previous vessel-rupture studies have concentrated on a specific set of exposure conditions, there is a need for more comprehensive parametric studies.

**Methods:**

Probability of rupture and rupture times were measured by exposing the large blood vessel of a live earthworm to high-intensity focused ultrasound pulse trains of various characteristics. Pressures generated by the ultrasound transducers were estimated through numerical solutions to the KZK (Khokhlov-Zabolotskaya-Kuznetsov) equation. Three ultrasound frequencies (1.1, 2.5, and 3.3 MHz) were considered, as were three pulse repetition frequencies (1, 3, and 10 Hz), and two duty factors (0.0001, 0.001). The pressures produced ranged from 4 to 18 MPa. Exposures of up to 10 min in duration were employed. Trials were repeated an average of 11 times.

**Results:**

No trends as a function of pulse repetition rate were identifiable, for either probability of rupture or rupture time. Rupture time was found to be a strong function of duty factor at the lower pressures; at 1.1 MHz the rupture time was an order of magnitude lower for the 0.001 duty factor than the 0.0001. At moderate pressures, the difference between the duty factors was less, and there was essentially no difference between duty factors at the highest pressure. Probability of rupture was not found to be a strong function of duty factor. Rupture thresholds were about 4 MPa for the 1.1 MHz frequency, 7 MPa at 3.3 MHz, and 11 MPa for the 2.5 MHz, though the pressure value at 2.5 MHz frequency will likely be reduced when steep-angle corrections are accounted for in the KZK model used to estimate pressures. Mechanical index provided a better collapse of the data (less separation of the curves pertaining to the different frequencies) than peak negative pressure, for both probability of rupture and rupture time.

**Conclusion:**

The results provide a database with which investigations in more complex animal models can be compared, potentially establishing trends by which bioeffects in human vessels can be estimated.

## Background

In the evaluation of new high-intensity therapeutic ultrasound (HITU) procedures, such as vessel occlusion, sonothrombolysis, brain-tumor ablation, and blood-brain-barrier opening, an important safety consideration is the integrity of the vasculature during the procedure. This is particularly true of transcranial procedures, where consequences of vessel rupture are likely to be severe. Whether the vessels are targeted as part of the procedure, or reside in the proximity of the target location, it is critical to know the likelihood that the vessels will rupture under the exposure conditions existing in the procedure.

Hynynen et al. [1] studied vessel constriction and rupture in a rabbit model, using 1.5 MHz HIFU (high-intensity focused ultrasound) at intensities between 4400 and 8800 W/cm^2^. They found that 1-second exposures caused the vessels to constrict at all intensity levels. At intensities above 5800 W/cm^2^, rupture occurred in 5 of the 23 vessels sonicated. Using an array of exposures (2 rows of 4 sonications surrounding the vessel), Rivens et al. [2] found that 2-second exposures at 4660 W/cm^2^ produced hemorrhage in 8 of 10 rat femoral arteries.

Hoerig et al. [3] examined vessel rupture using explanted porcine femoral arteries, as part of a study into whether rupture could be predicted using passive cavitation detection. They sonicated vessels using a 3.3 MHz ultrasound transducer that produced peak negative pressures (PNP’s) between 10.9 and 12.5 MPa, using a 50% duty cycle and exposures up to 5 min long. For PNP’s above about 12 MPa, vessel rupture occurred within 5 min for all trials.

The above studies have identified thresholds for vessel rupture in terms of PNP, or intensity. As the investigations were primarily motivated by a particular procedure such as vessel occlusion, they did not involve a wide range of exposure conditions. From a safety perspective, it is also important to know quantitatively the sensitivity of the vessel-rupture thresholds to sonication parameters, including frequency, pulse repetition rate (PRF), duty factor (DF), and exposure duration. Parametric studies spanning the ranges of interest of these variables are challenging, owing to the large number of vessels that must be exposed, and the lengthy exposure times that can be involved in defining the edges of the safety envelope. Hence, it is worthwhile exploring simple animal models that can be obtained in large numbers at low cost.

In the present investigation, a parametric study into the effect of sonication parameters on the threshold for rupture was conducted, using a *lumbricus terrestris* (earthworm) model. Elmer and Palmer [4] used the oxygen-carrying protein filling the earthworm vessels as a blood-cell substitute. Wahab et al. [5] used earthworms in a study of the effects of high-intensity focused ultrasound on nerve functionality. The earthworm vasculature, while limited in its ability to mimic that of a human, possesses basic properties important for a vascular model. The vessel diameter, on the order of a few hundred microns, is between the smallest capillaries (arterioles) in the human cerebral vasculature and the largest. The vessels contain a blood-like fluid flowing under pressure, which rapidly spreads upon vessel rupture. The vessels are embedded in soft tissue, which is useful for simulating acoustic-propagation conditions in humans. Still, since the connection of the earthworm vasculature to that of mammals has not been established, the goal is not to translate the results of this study directly to HITU procedures in humans. Rather, this study provide a foundational investigation into the sensitivity of vessel rupture to the parameters characterizing HITU procedures; future investigations in more complex models can leverage the results of this study to generate results more directly translatable to procedures performed on humans. This translation issue is discussed further in Discussion section.

In the study, three frequencies (1.1, 2.5, and 3.3 MHz) were considered, as were three PRFs (1, 3, and 10 Hz), and two DFs (0.0001, 0.001). The pressures produced ranged from 4 to 18 MPa. Exposures of up to 10 min in duration were employed. The experiments were repeated an average of 11 times.

## Methods

**Fig. 1:**
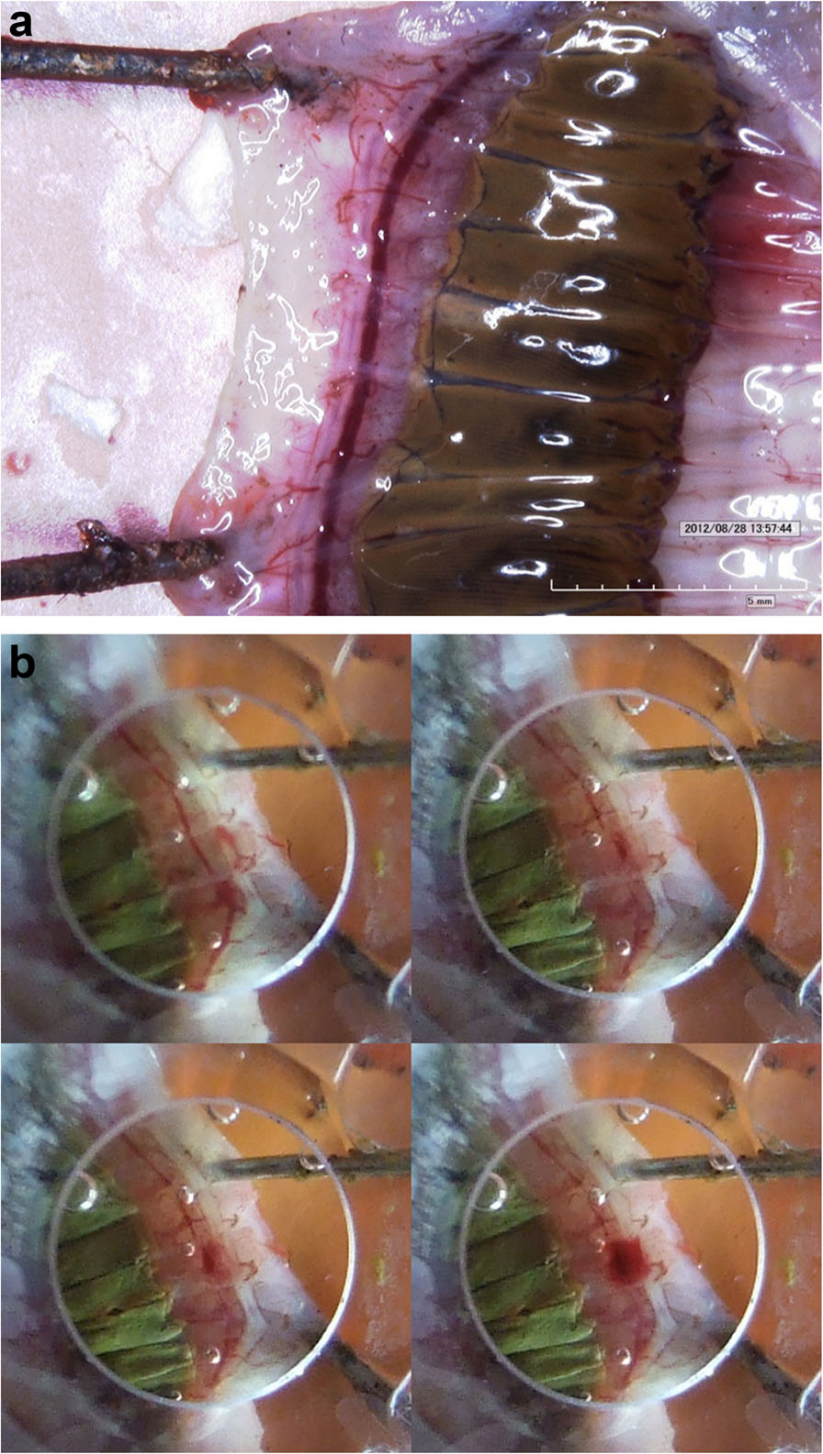
**a** Earthworm dissected to expose the ventral blood vessel. Nerve cord is also visible on the left side of the blood vessel. **b** Image of earthworm vessel shortly after rupture

Earthworms 10–15 cm long were purchased from a local fishing bait store to use in the experiments. The earthworms were kept in a container with damp soil and stored in a refrigerator at 4 °C, which allowed storage for 6 to 7 weeks, though earthworms were typically sonicated within days after purchase. The earthworms were anaesthetized by submerging them in a 10% ethyl alcohol (90% water) mixture at room temperature for about 20–25 min prior to the experiments. Once the earthworm was properly anaesthetized, a 3-cm-long cut along the anteroposterior axis was made on the skin, halfway between the two ends of the worm. The cut was made on the side of the body, so as to not cause damage on the ventral blood vessel, but to fully expose it (Fig. 1a). During the procedure, the earthworms were secured with surgical pins to an ultrasound-absorbing rubber plate, to reduce any remaining motion, which could produce targeting errors.

Two different transducers were used to transmit acoustic energy to the target area of the earthworm. The first, a HIFU transducer (H-102, Sonic Concepts Inc., Bothell, WA) with a 20 mm central opening for imaging, has a geometric focus of 6.3 cm and a diameter of 6.4 cm. The transducer was used at both the 1.1 MHz fundamental frequency and the 3.3 MHz third harmonic. A second HIFU transducer (H-147, Sonic Concepts Inc., Bothell, WA), having a geometric focus of 5.0 cm, a 23 mm imaging opening, and a diameter of 6.0 cm, was operated at the fundamental frequency, 2.5 MHz. Thus, between the two transducers, frequencies of 1.1, 2.5, and 3.3 MHz were used in the study. The energy emanating from the transducer was coupled to the worm using a coupling cone filled with tap water. In the experiments, the HIFU transducers were driven by signals generated by a function generator (AFG-3012, Tektronix, Beaverton, OR) through a 55 dB RF power amplifier (A-300, E&I, Rochester, NY). DFs of 0.001 and 0.0001 and PRFs of 1, 3, and 10 Hz were considered at the 3.3 MHz frequency. It was found during the experiments at 3.3 MHz that the results were not strongly dependent upon PRF. Hence, to reduce the experimental effort, at 2.5 and 1.1 MHz the PRF was held at 1 Hz, except for one set of experiments performed with a PRF of 10 Hz. Driving voltages at the function generator ranged from 100 to 450 mV. The acoustic powers corresponding to these driving voltages were measured using a radiation-force balance (Ohmic Instruments, Easton, MD, USA). The focal pressures corresponding to these powers were estimated using the HIFU_Simulator [6] beam propagation code. The different driving voltages and acoustic powers and pressures are shown in Table 1.

**Table 1:** Sonication characteristics for the three transducers used in the study

Transducer frequency (MHz)	Measured driving voltage (*V*, peak-peak)	Computed acoustic power at transducer (*W*)	Computed peak negative pressure (MPa)
1.1	84	12.77	4.21
	121	26.93	5.83
	154	44.10	7.14
	188	66.30	8.40
	223	94.00	9.62
	256	124.64	10.69
2.5	113	24.72	11.22
	143	39.95	13.25
	176	61.00	15.19
	202	80.78	16.56
	224	99.74	17.64
	239	113.82	18.35
3.3	114	11.25	8.50
	144	18.08	9.89
	184	29.77	11.36
	224	44.40	12.54
	254	57.32	13.29
	274	66.87	13.74
	280	69.88	13.87
	304	82.60	14.36

In each experiment, a layer of ultrasound gel (Aquasonic 100, Parker Laboratories, Inc., Fairfield, NJ) was placed upon the vessel of the worm, and the orifice of the coupling cone was positioned about 3 mm above the vessel. The intact status of the blood vessel was initially confirmed from visual inspection from the top of the transducer, as shown in Fig. 1a. Sonication of the vessel was then performed, for a duration of up to 10 min, until the vessel could be seen to burst. Bursting was defined by a spreading of blood well beyond the vessel wall, as shown in Fig. 1b. For each set of sonication conditions used, the rupture time was recorded. When bursting did not occur within 10 min of sonication, a value of 10 min was entered as the time. As discussed further in the final section, assignment of the artificial 10-minute time was an effort to incorporate quantitatively the extremely long exposure time. The 10-minute limit on rupture time primarily affected the data at the two lowest pressure levels in Table 1, for all three frequencies. In the statistical analysis performed to discern possible differences between rupture-times based upon different parameters, tests were not performed on data sets that included the artificial 10-minute rupture times. For the other data sets, unpaired, two-tailed *t* tests were performed to compare rupture times for different conditions.

Uncertainty in the rupture-time measurement was dictated primarily by how fast the blood emerged from the rupture (Fig. 1b.) Typically, a few seconds was required to confirm that a rupture had occurred. In some cases, the spread of blood was slow, possibly due to low volume of blood in that particular subject, or possibly because of a small hole. In any event, on rare occasions as many as 10 s could be required to confirm rupture. Once the protocol was established (and all observers were trained on it), particularly the distance above the worm that the orifice of the coupling cone was placed, variability between observers was small, on the order of a few seconds. Both the interobserver and intraobserver variability were small compared to the subject-to-subject variability, which, as measured by the standard deviation of the measured rupture times, often exceeded 100 s.

The number of repeat trials for a given set of sonication parameters varied, depending in part upon the availability of worms. The average number of repeats was 11, with the smallest being 6, and the largest 20. A total of approximately 800 experiments were performed.

## Results

### Effect of pulse repetition frequency

**Fig. 2:**
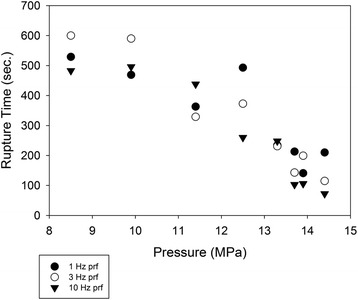
Rupture time as a function of peak negative pressure, for different pulse-repetition frequencies. Ultrasound frequency = 3.3 MHz

For the exposures at the 3.3 MHz frequency, an unpaired, two-tailed *t* test was performed to compare rupture times for different PRFs, at the same duty factor and the same pressure. While rupture times for some pairs of PRFs considered were found to be statistically different (*p* = .05), only at the highest pressure level (14.4) and 0.001 DF were all 3 rupture times different. No identifiable trend with PRF emerged at either DF. Rupture-time values for a PRF of 0.0001 are shown in Fig. 2. The PRF producing the highest rupture time at a given pressure varies considerably from pressure to pressure, suggesting that there is no clear dependence of rupture time upon PRF.

### Effect of duty factor

**Fig. 3:**
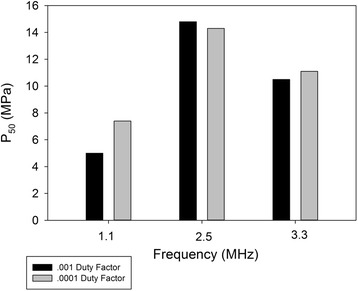
Peak negative pressures at which rupture occurred 50% of the time, for each of the frequencies and duty factors

**Fig. 4:**
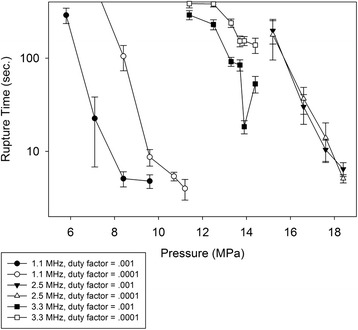
Rupture time as a function of peak negative pressure, for all frequencies and both duty factors

Figure 3 displays the pressure P_50_ at which the probability of rupture reached 50% . P_50_ was not a strong function of duty factor at the 2.5 and 3.3 MHz frequencies. At 1.1 MHz, the pressure corresponding to 50% rupture for the .001 DF was about 1.5 times that for the 0.0001 DF. In Fig. 3, all PRFs were combined. Rupture-times, plotted in Fig. 4, were a strong function of DF at the 1.1 and 3.3 MHz frequencies, but almost independent of DF at 2.5 MHz. As examples, consider the pressures 8.4, 13.2, and 16.6 MPa. As will be seen shortly, these values represent the approximate threshold values for rupture 100% of the time for the 1.1, 3.3, and 2.5 MHz transducers. At the 8.4 MPa pressure, the rupture time was 5.1 s for the 0.001 DF and 105 s for the 0.0001 DF. The difference was found to be highly significant (*p* < .01) in a two-tailed *t* test. At 13.2 MPa, the rupture time was 91.8 s for the 0.001 DF and 238 s for the 0.0001 DF. The difference was also found to be highly significant (*p* < 0.001). At 16.6 MPa, the rupture times were 30.1 s (DF = 0.001) and 36.8 s (DF = 0.0001); these values were not found to be statistically significant.

### Effect of pressure

**Fig. 5:**
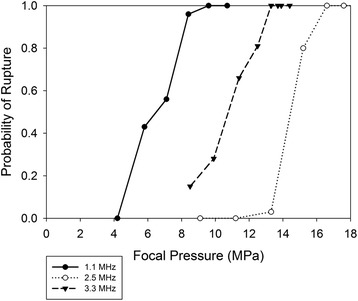
Probability of rupture as a function of peak negative pressure, for the three different ultrasound frequencies. Data from all pulse repetition frequencies and duty factors are included

Returning to Fig. 4, it can be seen that, for a given frequency, the rupture time decreases rapidly with increasing pressure. At 1.1 MHz, for example, the rupture time decreases by an order of magnitude when the peak negative pressure changes from 6 to 7 MPa. The sensitivity of rupture time to pressure was found to be less intense at 3.3 MHz than 1.1 MHz and 2.5 MHz, though a decrease with increasing pressure was still evident. As probability of rupture was not found to be a strong function of DF, the results for different DFs were combined and plotted as a function of pressure in Fig. 5. The pressure interval over which the probability of rupture increases from 0 to 1 is roughly 4 MPa for all three frequencies. Of note is the fact that the pressure thresholds for the 2.5 MHz frequency exceed those for the 3.3 MHz frequency. This issue is addressed in the Discussion section.

### Correlation with mechanical index

**Fig. 6:**
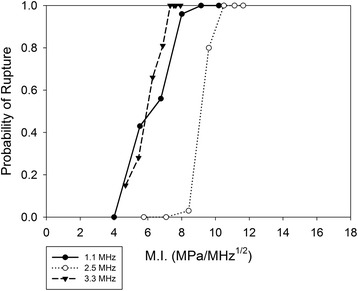
Probability of rupture as a function of mechanical index, for the three different ultrasound frequencies. Data from all pulse repetition frequencies and duty factors are included

**Fig. 7:**
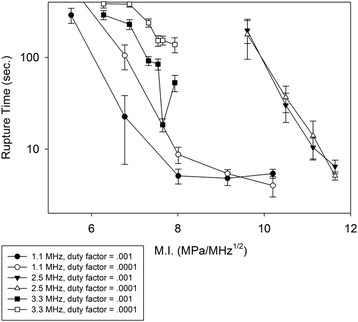
Rupture time as a function of mechanical index, for all frequencies and both duty factors

In an attempt to combine the effects of frequency and pressure into a single independent variable, the data of Fig. 5 were replotted as a function of the mechanical index, given by

1 $$ \mathrm{M}\mathrm{I}={P}_{-}/\sqrt{f} $$

where *P*_*−*_ is the peak negative pressure and *f* the center frequency. The results are shown in Fig. 6. When plotted as a function of PNP (Fig. 5), the spacing of the leftmost and rightmost curves, measured at the 50% probability line, is about 8.3 MPa, or about 80% of the average (10.5 MPa) of the three 50% pressures. That is, the width of the profile is about 80% of the mean value of the independent variable. For the MI (Fig. 6), less of a spread occurs. The width (3 MPa/Hz^1/2^) is approximately 50% of the mean value (3 MPa/Hz^1/2^) of the independent variable. In addition to rupture probability, an attempt was made to correlate rupture time as a function of MI. Results are plotted in Fig. 7. If we consider a rupture time of 100 s, the width between the leftmost curve in Fig. 7 (1.1 MHz, .001 DF) and the rightmost curve (2.5 MHz, both DFs), the width is approximately 4 MPa/MHz^1/2^, or about 50% of the mean (8 MPa/MHz^1/2^) M.I. corresponding to a 100 s rupture time for the 6 curves. (a slight downward extrapolation is required to reach the 100-second rupture time for the 3.3 MHz/0.001 duty factor curve.) In other words, the width of the profile is approximately half of the mean. For the correlation of rupture time with pressure (Fig. 4), the width measured at the 100-second rupture time is about 9.4 MPa, or about 75% of the mean (12.5 MPa) pressure corresponding to the 100-second rupture time for the 6 curves. (again, a slight downward extrapolation is required to reach the 100-second rupture time for the 3.3 MHz/0.001 DF curve).

## Discussion

The observation that DF only weakly affects probability of rupture (e.g., at most a 50% change in *P*_50_ in Fig. 3), but substantially affects rupture time (e.g., a factor of 10 difference in rupture time at 8.5 MPa in Fig. 4), may be an evidence that inertial cavitation initiation is a prerequisite for rupture [7], but that the complete rupture process involves bubble action over time. The cavitation initiation would involve a strong dependence on pressure but not much on DF, while bubble action would have more effect for longer duty cycles. Given the presumed role of cavitation, it is somewhat surprising that the pressure threshold for rupture is lower at 3.3 than 2.5 MHz. However, to a significant extent, this unintuitive result is an artifact of the pressure determination, in the following sense.

Focal pressures were estimated using beam propagation modeling, through the HIFU_Simulator software, which is based upon the KZK equation. The accuracy of the KZK model reduces as the propagation angle steepens, particularly as the transducer *f* number decreases to 1 or less. The *f* number was about 1.0 for the 1.1 and 3.3 MHz experiments, and 0.8 for the 2.5 MHz. Soneson [8] provides criteria for estimating errors in the KZK model arising from steep-angle propagation. For the characteristics of the present study, the pressure is overestimated by roughly 10% for the 1.1 MHz frequency, 20% for 3.3 MHz, and 40% for 2.5 MHz. As precise steep-angle corrections to KZK are implemented in the HIFU_Simulator software, the data will be replotted in terms of the modified pressures. For now, we remark that decreasing the 2.5 MHz pressures around 40% and the 3.3 MHz pressures around 20% places the curves (Figs. 4 and 5) for the two pressures in close proximity.

For experiments at low pressure, where ruptures occurred in only some trials, a value of 10 min was entered for trials where no rupture was observed in 10 min. This practice was performed only on data sets where rupture occurred at least 50% of the time. The use of the 10-minute limiting value enabled the data from all trials at a given pressure to be used in identifying rupture-time trends as different parameters were varied. Ignoring the trials where no rupture was observed would artificially reduce the average rupture time for that pressure, since the values ignored exceeded 10 min, perhaps by a large (even infinite) amount. Entering the value of 10 min also skewed the average downward to some extent, but the presence of the other values having rupture times less than 10 min mitigated the effect to some extent. The main consequence of bounding the rupture time at 10 min is the flattening of the data for the two lowest pressures (11.4 and 12.5 MPa) at the 3.3 MHz frequencies in Fig. 4, as well as the corresponding points in Fig. 7. In reality, these curves would be steeper (decreasing faster from left to right) at the low pressures/MI.

The vessel-rupture experiments of Hoerig et al. [3] were performed using the same type of transducer used in this study for the 3.3 MHz experiments. The peak-negative pressures were also estimated in a similar manner, using the HIFU_Simulator (Soneson 2009) software. Hence, though the experiments of Hoerig et al. were performed at a much higher DF–50% vs 0.1 and 0.01% for the present study—some comparisons can be made. Several observations are noteworthy:

1) The standard deviations of the measurements are typically comparable to (and slightly smaller than) the mean rupture times in the experiments of Hoerig et al. The same was observed in the present study. In the figures, we plot standard error of the mean on a logarithmic scale, making extraction of the standard deviation difficult, so as examples we examine the rupture times for 3.3 MHz exposure with a 0.001 DF. The mean rupture time for 13.3 MPa exposure was 92 s, with a standard deviation of 65 s. At 13.9 MPa, the mean rupture time was 18.4 s, with a standard deviation of 17.4 s. The large uncertainties are indicative of large subject-to-subject variation occurring in biological tissues. In the future, further categorization of subjects by factors such as vessel diameter or vessel stiffness (as measured by a mechanical tensile test performed on a segment of the vessel away from the rupture location) would be highly worthwhile.

2) Hoerig et al. present results for PNP’s of 10.9 to 12.5 MPa. For the pressure at which the most samples are available (*n* = 6), 12.1 MPa, a mean rupture time of 57 s (standard deviation of 40) was observed. In our study, the mean rupture time for 12.1 MPa for the higher DF (0.1%) was approximately 250 s (Fig. 3), with a standard deviation of 200 s. For the 3.3 MHz frequency, the average reduction in rupture time going from a DF of 0.0001 to 0.001 was 40% (factor of 0.6). Crudely applying the 0.6 reduction factor two more times to achieve the same order of magnitude DF used by Hoerig et al., we find that the rupture times in our study are on the order of 100 s (standard deviation ~ 70 s.) The rupture times for our study and that of Hoerig et al. are comparable. (a *t* test yields no statistically significant difference.)

3) In going from a PNP of 10.9 to 12.5 MPa, Hoerig et al. found a reduction in rupture time of about 32% (81 to 55 s), though quantitative estimates must be viewed with caution, as the number of samples is small (*n* = 2 and *n* = 3) at both pressures. Over a comparable range, from 11.4 to 13 MPa (11.4 MPa being the smallest pressure where data is available in our study), we measured a reduction in rupture time of about 50% (290 to 144 s) at the 0.001 DF and 24% (384 to 291 s) at the 0.0001 DF. We conclude that the variability in rupture time, the actual rupture time (crudely adjusted for different DFs), and change in rupture time with pressure, agree in a semi-quantitative manner between the investigation of Hoerig et al. and the present one.

One measure that demonstrates more of a difference between the two studies is the threshold for rupture. Hoerig et al. noted that no vessel ruptures were observed for the three trials performed for a PNP less than 10.9 MPa. From Fig. 4, the probability of rupture for the earthworm vessels drops to zero at around 7 MPa for 3.3 MHz sonication. As the probability of rupture was not found to be a strong function of DF (though this conclusion was based upon the limited DF range considered), the threshold of rupture may be higher for porcine arteries than earthworm vessels. The use of degassed saline as a blood surrogate by Hoerig et al., compared with native blood in the earthworm study, may have been a factor in initiating intravessel cavitation and subsequently vessel rupture. The difference in vessel size (roughly 3 mm diameter for swine vessels, 0.3 mm for earthworms), and the presence of fluid pumping through the swine vessels are other factors to consider, though these elements would more likely play a role if thermal effects were significant. The very low DFs in our study were maintained to deliberately minimize thermal effects. We note that in one set of 10-minute exposures performed at 3.3 MHz and a transducer driving voltage that produced a focal pressure of about 10 MPa, the temperature rise measured by a thermocouple near the focus was repeatedly between 1 and 2°. This increase in temperature was judged too low to influence vessel rupture.

Mechanical index produces a degree of collapse of the data (Fig. 5 vs Fig. 6; Fig. 4 vs Fig. 7), but does not provide enough collapse to allow prediction based upon a single independent variable. It is worth considering whether a frequency exponent different from 0.5 in the MI will produce a better collapse of the data. Increasing the exponent would have the salutary effect of moving the 2.5 MHz curve toward the 1.1 MHz. However, given that the top half of the 3.3 MHz curve is already furthest to left in Fig. 6, anything but a small increase in the exponent would spread the curves apart. It is our conjecture that modifications to account for steep-angle propagation in the KZK model will produce enough changes to the pressures that the exponent of 0.5 in the standard definition of M.I. (Eq. 1) will be optimal, perhaps even allowing accurate predictions of rupture probability based on the knowledge of the single independent variable, M.I. For rupture times correlated with M.I. (Fig. 7), a higher exponent appears to be beneficial (further shifts to the left of the higher-frequency curves); a value between 0.8 and 0.9 seems to provide the best correlation. With adjustment of the KZK pressures to account for steep-angle propagation, this value will likely decrease from 0.6 to 0.7.

It is important to place the large amount of data generated in this study in its proper perspective relative to HITU procedures involving human vasculature. As acknowledged in the Introduction, the direct translational value of the results is limited, i.e., for the most part, they cannot be used to define safety thresholds in humans without supplementary information from more realistic models. One important use of the present study is to create an awareness of the potential sensitivity of HITU procedures to important exposure parameters. For example, our results show that the rupture time is highly sensitive to duty factor for exposure at 1.1 MHz. While the expected vessel rupture times for a therapeutic ultrasound procedure on a human cannot be taken directly from our results, it will hopefully now be recognized that rupture times based upon the duty factor of 1% should not be used for treatment planning of a clinical procedure employing a duty factor of, say, 10%.

The study can also serve as a foundation for studies in animals that are closer to humans. The optimal way to perform investigations on this type is to calculate ahead of time the sample size for the study design. Our results provide an estimate for the likelihood of rupture occurring under different circumstances, and a measure of subject-to-subject variation, and can aid in the determination of sample size for follow-up studies. As an example, our conclusion that rupture thresholds are relatively insensitive to pulse repetition frequency could help reduce the scope of more realistic but more expensive animal trials.

In addition to being a foundational study for further work, the present investigation is part of a database of animal studies that can inform decisions on procedures in humans. All of the animal studies have limited translational capacity. The study by Hoerig et al. involves an animal model (porcine) that is closer to human than the worm model, but also involves degassed saline as a blood substitute, in an excised vessel that is no longer part of a living being. In aggregate, all of these limited studies provide a scientific base upon which predictions in humans can be made, especially if a consensus can be formulated from all of the limited animal models. Above we argue that there is a considerable overlap between the very different models of Hoerig et al. and ourselves, especially once adjustment is made for the different duty factors. For example, Hoerig et al. show a rapid increase in rupture probability occurring around a pressure of 12 MPa (at 3.3 MHz frequency), while we show a rapid increase at around 8 MPa, at a much lower duty factor. This range from 8 MPa (again, 8 MPa being conservatively low due to the very low duty cycle) to 12 MPa provides useful information for establishing safety thresholds for therapeutic ultrasound procedures in humans. Hence, especially if additional confirmation is provided from subsequent animal models, a safety threshold in the range of 8 to 12 MPa (possibly with an additional factor of safety) might be appropriate for 3.3 MHz exposures.

As a final perspective of the translational value of this work, we note that some of our results address more the physics of therapeutic ultrasound exposure than the features of the animal model. For example, the results on whether a mechanical index (M.I.) yields a better correlation of the rupture data than peak negative pressure, and what the exponent of the frequency dependence should be, could very well carry over to the human condition, as M.I. involves the physics of cavitation in soft-tissue media.

The results of this study are most relevant to procedures not involving microbubbles, or other agents such as nanoparticles [9], that can reduce the required level of ultrasound intensity. The pressures considered in the study likely exceed those in most sonothrombolysis or blood-brain-barrier procedures employing enhancing agents. The results in the current investigation pertain, for example, to HITU ablation, vessel cauterization, or clot-lysis procedures not employing microbubbles. Vessel rupture in these procedures could occur due to the presence of sufficiently high intensity outside the target volume, or to targeting errors.

## Conclusions

This study examined HIFU-induced vessel rupture in an earthworm model, using ultrasound frequencies of 1.1, 2.5, and 3.5 MHz. No trends as a function of pulse repetition rate were identifiable, for either probability of rupture or rupture time. Rupture time was found to be a strong function of DF at the lower pressures; at 1.1 MHz the rupture time was an order of magnitude lower for the 0.001 DF than the 0.0001. At moderate pressures (and 3.3 MHz frequency), the difference between the DFs was less and nearly non-existent at the highest pressure (2.5 MHz frequency). Probability of rupture was not found to be a strong function of DF. Rupture thresholds (non-zero probability of rupture) were about 4 MPa for the 1.1 MHz frequency, 7 MPa at 3.3 MHz, and 11 MPa for the 2.5 MHz, though the pressure value at 2.5 MHz frequency will likely be reduced when steep-angle corrections are accounted for in the KZK model used to estimate pressures. MI provided a better collapse of the data (less separation of the curves for different frequencies) than PNP, for both probability of rupture and rupture time. The results of this study constitute a database against which investigations in more complex animal models can be compared, potentially establishing trends by which bioeffects in human vessels can be estimated.

## Abbreviations

DF: Duty factor
HIFU: High-intensity focused ultrasound
KZK: Khokhlov-Zabolotskaya-Kuznetsov
MI: Mechanical index
PNP: Peak negative pressure
PRF: Pulse repetition rate
